# The effect of polytetrafluoroethylene particle size on the properties of biodegradable poly(butylene succinate)-based composites

**DOI:** 10.1038/s41598-021-86307-x

**Published:** 2021-03-24

**Authors:** Shudong Chen, Xiangfang Peng, Lihong Geng, Hankun Wang, Jialin Lin, Binyi Chen, An Huang

**Affiliations:** 1 Key Laboratory of Polymer Materials and Products, School of Materials Science and Engineering, Fujian University of Technology, Fuzhou, 350118 China; 2 Department of Biomaterials, International Center for Bamboo and Rattan, Beijing, 100102 China

**Keywords:** Biomaterials, Environmental sciences

## Abstract

Poly(butylene succinate) (PBS)/polytetrafluoroethylene (PTFE) composites, including three types of PTFE powders, were prepared by melt blending using a HAAKE torque rheometer. Microcellular foams were successfully fabricated by batch foaming with supercritical fluids (scCO_2_). The effects of PTFE powder type on crystallization, rheological properties and foaming behavior were studied. PTFE L-5 and PTFE JH-220 powders showed good dispersion in the PBS matrix, and PTFE FA-500 powder underwent fibrillation during the melt blending process. All three PTFE powders gradually increased the crystallization temperature of PBS from 78.2 to 91.8 ℃ and the crystallinity from 45.6 to 61.7% without apparent changes in the crystal structure. Rheological results revealed that PBS/PTFE composites had a higher storage modulus, loss modulus, and complex viscosity than those of pure PBS. In particular, the complex viscosity of the PBS/P500 composite increased by an order of magnitude in the low-frequency region. The foamed structure of PBS was obviously improved by adding PTFE powder, and the effect of fibrillated PTFE FA-500 was the most remarkable, with a pore mean diameter of 5.46 μm and a pore density of 1.86 × 10^9^ cells/cm^3^ (neat PBS foam: 32.49 μm and 1.95 × 10^7^ cells/cm^3^). Moreover, PBS/P500 foam always guarantees hydrophobicity.

## Introduction

Polymer foams are generally considered to be gas-filled composites, which are characterized by light weight, low density, shock absorption and excellent mechanical properties. They can realize lightweight polymer materials without losing or while even improving the performance of materials^1^. Many types of polymers^2–5^ can form foams, such as polystyrene (PS), polyvinyl chloride (PVC), polypropylene (PP), and polyethylene (PE). In recent decades, traditional petroleum polymers have primarily been used, which are difficult to recycle and degrade in nature. Because of the increasing concern of sustainable development and the effect of petrochemicals on the environment, biodegradable polymers have attracted much attention. As one of the biodegradable plastics with excellent comprehensive performance, poly(butylene succinate) (PBS)^6–9^ has good biodegradability, processability, heat resistance and chemical resistance, as well as mechanical properties similar to those of PP and PE. However, PBS is a polycondensation product with a low molecular weight and highly linear molecular structure, resulting in a low melt strength. When fabricating PBS foams via supercritical carbon dioxide (sc‐CO_2_), the melt strength is too low to support the cells during cell growth, causing large cell sizes and collapsed cells^10^, which limits their wide application. The method of enhancing the melt strength or elastic modulus is the key to developing high-performance PBS foams, which can further broaden their application areas.

Recently, chemical modification has been used as an alternative for improving PBS melt strength. Zhou et al.^11^ improved the viscoelasticity and foamability of PBS through a chain extender (CE). A cross-linking structure was formed when the CE content was 0.75 phr, and the expansion volume ratio of the foam increased almost 15-fold. Li et al.^12^ also prepared high viscosity PBS with dicumyl peroxide (DCP) as a crosslinking agent. Boonprasertpoh et al.^13^ enhanced the viscosity of PBS by crosslinking with DCP or branching with N3300 (branching agent (0–4 phr)), resulting in a higher degree of crosslinking, less crystallinity, and higher viscosity with increasing DCP and N3300. However, crosslinking also has disadvantages, such as chemical reagent residues and environmental and human health risks, as well as a lack of reprocessability and recyclability.

Adding nanofillers is a more common method for improving the melt strength of PBS. Chen et al.^14^ successfully fabricated lightweight, high-strength and electrically conductive PBS/carbon black (CB) nanocomposite foams with a density of 0.107–0.344 g/cm^3^ using a solid-state sc-CO_2_ foaming process. Lim et al.^15^ compared the performance of biodegradable PBS/carbon nanofiber (CNF) composites fabricated using three methods, specifically, solution blending, melt compounding, and solution and subsequent melt blending. Lim et al.^16^ also investigated PBS nanocomposites with multiwalled carbon nanotubes (MWCNTs) to study the effect of MWNTs on the physical characteristics of biodegradable PBS/CNT foams. Wu et al.^17^ prepared PBS/halloysite nanotube (HNT) nanocomposite foams by melt compounding and scCO_2_ foaming, indicating that HNTs obviously improved the foamability of the PBS matrix. However, these nanofillers easily aggregate during melt blending and are expensive, making them difficult to apply on a large scale in the preparation of high-performance foams.

More recently, virgin polytetrafluoroethylene (PTFE) powder has been used to improve the melt strength of polymers. Yang et al.^18^ prepared ultrahigh-cell-density polypropylene foam by scCO_2_ foaming in the presence of micro PTFE powders. Wang et al.^19^ reported a strategy for preparing lightweight and tough PP/PTFE nanocomposite parts with defect-free surfaces by combining in situ fibrillation and nanocellular injection molding technologies. Huang et al.^20^ prepared in situ tripolymer nanofibrillar composites (istp-NFCs) of Polylactic acid/Thermoplastic polyurethanes/PTFE (PLA/TPU/PTFE), investigating the effect of nanofibrillated PTFE on the ductility, crystallization, viscoelasticity and microcellular injection molding (MIM) of PLA/TPU blends. PTFE has attracted attention because of its high dispersion property, extremely high thermal stability, chemical resistance and extremely high melt viscosity property^21^. In addition, when foaming, the melt strength is usually too low to support the cells during cell growth for neat PBS, causing large cell sizes and collapsed cells. Adding fillers, such as CNFs and CNTs, is a common method. However, these fillers are expensive and easily agglomerate. As an alternative, PTFE has better dispersibility and a low price. It has a good heterogeneous nucleation effect, which can substantially improve the foaming nucleation of PBS. Moreover, PTFE has excellent stability, can be used at − 180 to 260 °C for a long time and does not affect the normal use of PBS as a biodegradable polymer. In addition, PTFE is a biocompatible polymer that is not harmful to the environment and humans.

We studied the synergistic effect of CNT and PTFE to achieve PBS functionalization in our previous research^22^. In contrast, this paper pays more attention to the modification of PBS by PTFE itself and different types of PTFE fillers, which better elucidates the influence of different types of PTFE on the PBS matrix and foaming process. We believe that different types of PTFE exhibit different properties in modification and melt processing; therefore, this work first fabricates lightweight PBS composite foams with low concentrations of different types of PTFE powder. A superior cell structure and low cell size were achieved. First, a series of PBS composites with three types of PTFE powder were fabricated by a direct melt-compounding approach. Then, the subsequent composite foams were prepared using a solid-state supercritical CO_2_ foaming process. The morphological characteristics and thermal, crystallization and rheological behavior of the solid PBS/PTFE composites, as well as their mechanical properties, were investigated. Then, the foaming behavior and mechanical properties of the foams, as well as their wettability, were analyzed. This study provides a convenient solution for improving the melt strength and foaming properties of PBS and expands the application prospects of PTFE as a filler. PBS/PTFE foams can be used in some fields, such as heat insulation, sound insulation and shock absorption. In addition, because PTFE has anti-drip characteristics, it can be used as a flame retardant material. As a biodegradable composite, the PBS matrix is eventually completely decomposed, leaving only a small amount of PTFE for recycling.

## Methods

### Materials

PBS (GS Pla) was acquired from Anqing He Xing Chemical Co., Ltd. (Anqing, China). PTFE (L-5, 5–10 μm) and PTFE (JH-220, 20–30 μm) were purchased from Shanghai Ganyi Plastic Technology Co., Ltd. (China) and PTFE (FA-500, 500 μm) were purchased from RiBen Dajin Co., Ltd. (China), which were named by P5, P220 and P500, respectively. Commercial CO_2_ (99.9%) was supplied by Fuzhou Xinhang Gas Co., Ltd. (Fuzhou, China).

### Preparation of PBS/PTFE composites

PBS pellets and three kinds of PTFE powder were dried at 80 ℃ for 2 h before blending. Then PBS/PTFE composites with different types of PTFE (3 wt% loading) were prepared by melt-blending using HAAKE internal mixer (Germany) with rotors speed 80 rpm at 140 ℃ for 10 min. The obtained composites were compression molded (rectangular sheet with a thickness of 1 mm) at 140 ℃ 20 MPa for 5 min. The samples were named as PBS, PBS/P5, PBS/P220, PBS/P500 based on PTFE type.

### Preparation of PBS/PTFE composite foams

**Figure 1:**
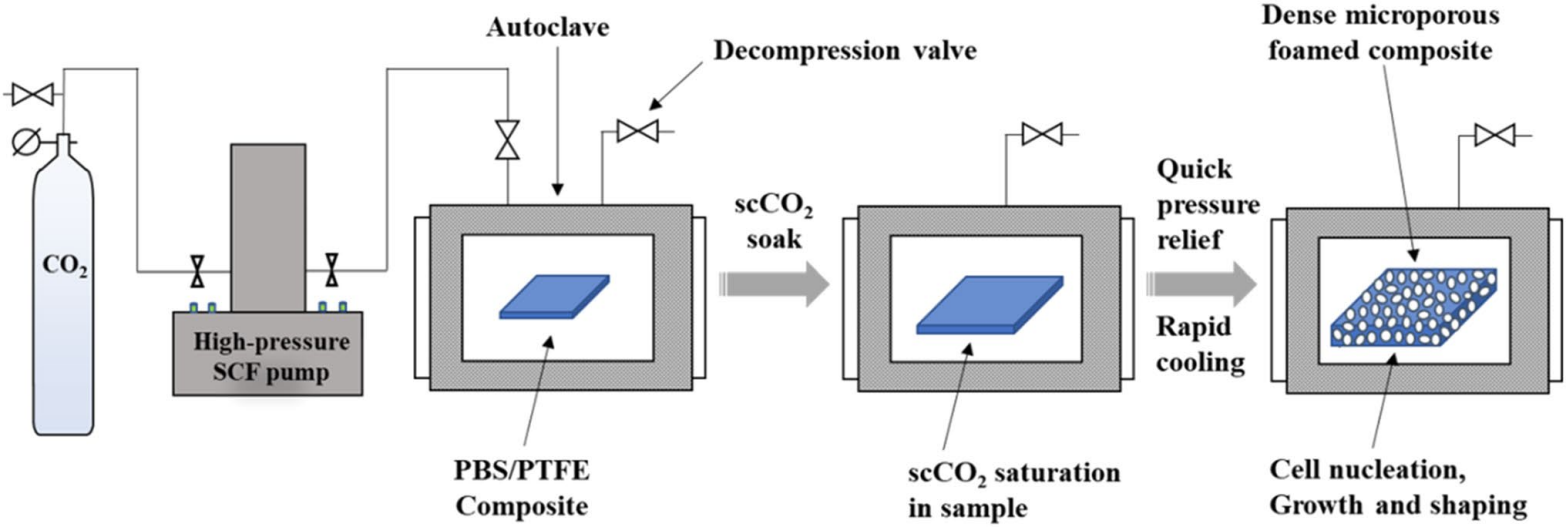
Schematic of batch foaming.

PBS/PTFE composite foams were fabricated via a solid-state supercritical CO_2_ foaming. Firstly, the composites were saturated with scCO_2_ in a high-pressure autoclave at 90 ℃ (below the melting point of PBS) and 20 MPa for 2 h to ensure equilibrium adsorption. And then the autoclave was rapidly depressurized and then cool down. The schematic of batch foaming is shown in Fig. 1.

### Characterization

#### X-ray diffraction (XRD)

XRD patterns of samples were obtained by X-ray diffractometer (XRD, Bruker D8, Germany) using CuKa radiation (40 kV and 20 mA) over a range of 10–50°, with a step interval of 4°/min.

#### Thermal characterization

The crystallization behavior of virgin PTFE, PBS and the composites were analyzed by differential scanning calorimetry (DSC 200 F3, Netzsch, Germany) in nitrogen atmosphere. Approximately 5–8 mg sample was used and first heated from room temperature to 150 ℃ and maintained 3 min to erase the thermal history. Then cooled down to 30 ℃ with cooling rate of 5 ℃/min and maintained 3 min, and then the temperature was raised again to 150 ℃ with heating rate of 5 ℃/min.

The crystallinities (*X*_*c*_) of neat PBS and the composites were evaluated using Eq. (1) as follows,

1 $${X}_{c}=\frac{\Delta {H}_{m}-\Delta {H}_{cc}}{\left(1-\varnothing \right)\Delta {H}_{m100}}\times 100\%$$

where Δ*H*_*m*_ is the melting enthalpy, Δ*H*_*cc*_ is the cold crystallization enthalpy, Δ*H*_*m100*_ is the melting enthalpy of polymer with 100% crystallization (PTFE: 82.0 J/g, PBS: 200.0 J/g), *φ* is the proportion of filler. Thermal degradation property was investigated using a thermo gravimetric analysis instrument (TG 209 F1 Libra, Germany) heating from room temperature to 600 °C with 10 °C/min in nitrogen atmosphere.

#### Mechanical properties

Mechanical properties of solid and foamed samples are measured with dumbbell samples (ASTM D638-03, type V), which solid samples were cut off from the hot-pressed sheet. The test was performed on the universal tensile machine (AGS-X, SHIMADZU, Japan) and the cross-head speed was set 5.0 mm/min. The average values reported were based on at least seven specimens.

#### Rheological test

Rheological measurement was carried out on the rotating rheometer (TA ARES G2, American) at 130 °C. 1.0% strain was set and the frequency varied from 100 to 0.01 Hz. At the same temperature and strain parameters, a time sweep of 1000 s duration was performed. The creep properties of the materials were tested at 130 °C with a constant stress of 100 Pa and duration of 1000 s.

#### Microstructure analysis

The fracture surface of solid composite and foams was observed by a field emission scanning electron microscope (FEI Nova Nano SEM 450, American) operating at 2 kV. All samples are fractured after being soaked in liquid nitrogen holding by two pliers for the SEM analysis and sputtered with gold to avoid electrification in the inspection. Pore sizes (D) were obtained by the Image-Pro Plus 6.0 software from the SEM images. Pore density (N_f_) was calculated by the equation^23^,

2 $${\text{N}}_{{\text{f}}} = \left[ \frac{N}{A} \right]^{\frac{3}{2}}$$

where N is the number of pores and A is the area of the image (cm^2^).

Foamed samples were cut into rectangles and porosities were obtained by weighing the samples and measuring their volume using the equation^24^

3 $$\mathrm{Porosity}=\frac{{V}_{f}{\rho }_{0}-{W}_{f}}{{V}_{f}{\rho }_{0}}\times 100\%$$

where *V*_f_ is the volume of the foamed sample, W_f_ is the measured weight of foamed sample, and *ρ*_*0*_ is the mass density of neat PBS.

The volume expansion ratio (Φ) of the foames was defined as the ratio of *ρ*_*s*_ (g/cm^3^) to that of *ρ*_*f*_^19^,

4 $$\Phi =\frac{{\rho }_{s}}{{\rho }_{f}}$$

where *ρ*_*s*_ and *ρ*_*f*_ are measured by water displacement method (ASTM D792) and represent the mass densities of the sample before and after foaming, respectively.

#### Water contact angle test (WAC)

The water contact angle (WAC) of the sample surface was measured by the contact angle measuring instrument (KRUSS DSA25, Germany). The samples were placed on the platform of the contact angle measuring instrument, and then the deionized water droplets were dropped on the surface of the foam sample by hanging drop method, each drop was 2 μL, and the corresponding static WCA value was measured. The WCA values after 20 min were also collected. The average value of the five values was calculated.

## Results and discussion

### Virgin PTFE

**Figure 2:**
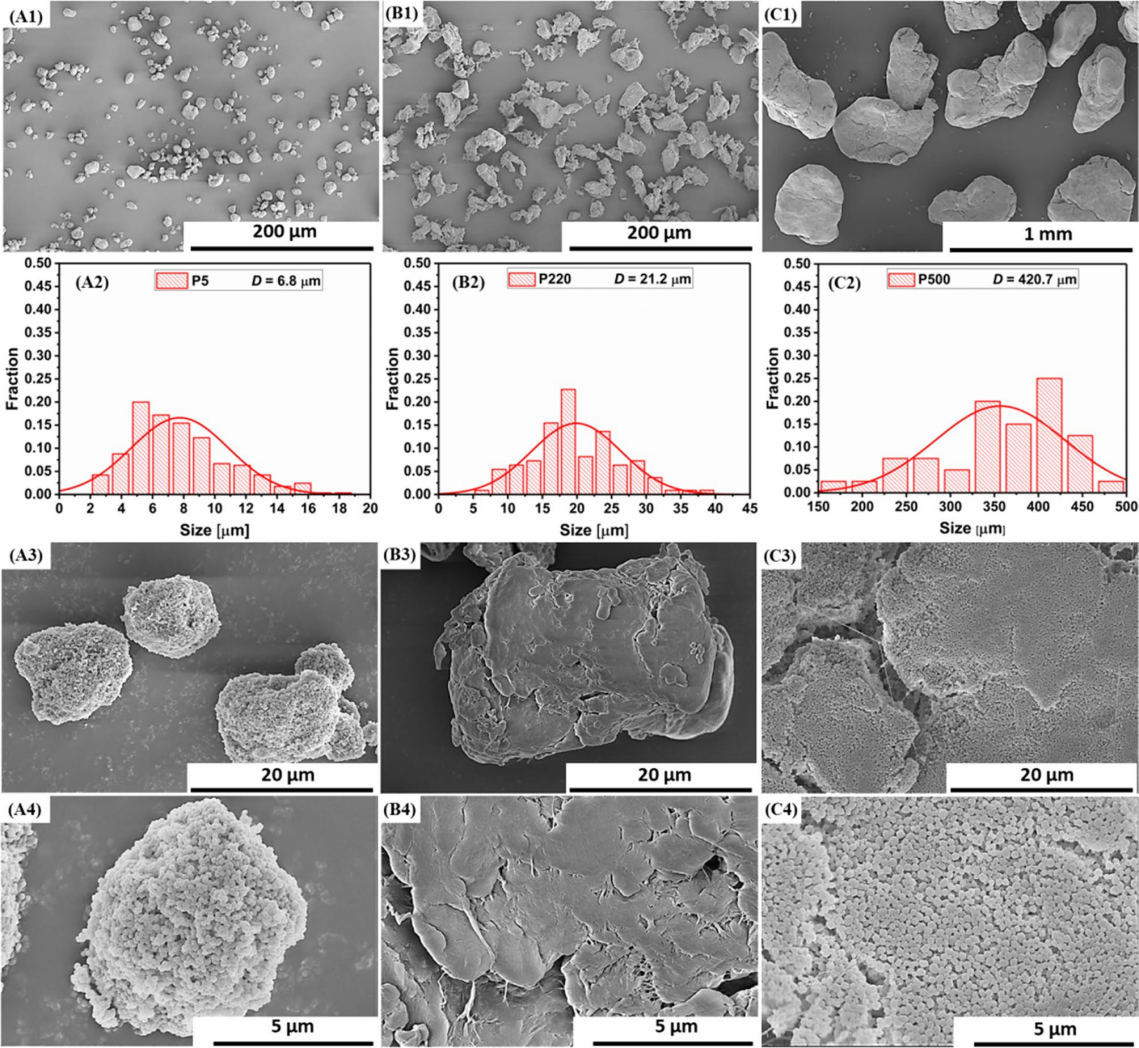
SEM micrographs and size distribution of PTFE powders: (**A**) P5, (**B**) P220, and (**C**) P500.

Figure 2 shows the SEM images of virgin PTFE and the particle size distribution obtained by counting particles in several SEM images using Image-Pro Plus 6.0 software. As shown in Fig. 2, the diameter of the P5 powder is between 5 and 10 μm, P220 powders have irregular shapes with an average diameter of 15–30 μm, and P500 powders have an average diameter of approximately 400 μm. Interestingly, at high magnification, Fig. 2A4 shows that P5 particles are clustered together by many smaller nanoparticles with nanogaps through intermolecular forces, which is similar to the structure of P500 powders (Fig. 2C4); however, P220 has a smooth surface.

**Figure 3:**
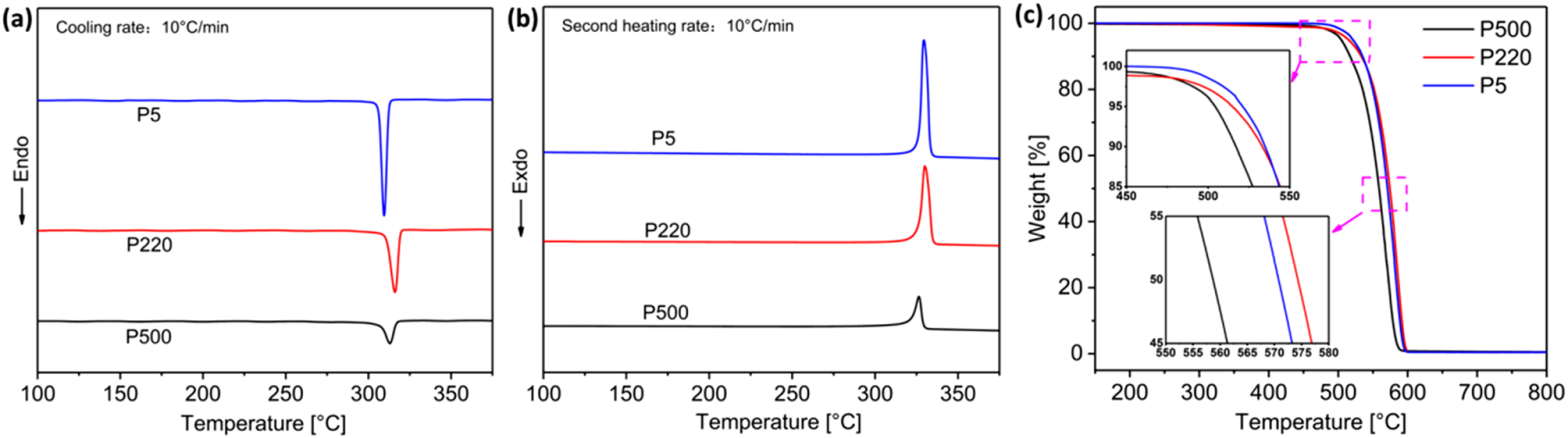
DSC curves of PTFE powders: (**a**) cooling, (**b**) second heating, and (**c**) TGA curves.

The thermal properties of virgin PTFE were determined using DSC and TGA. The curves and statistical data are shown in Fig. 3 and Table 1. In Fig. 3a, the crystallization temperature (T_c_) value is observed in the following order: P220 > P500 > P5, and all three types of PTFE have a high T_c_. Figure 3b shows that the melting temperature (T_m_) value is in the following sequence: P220 > P5 > P500, and all three PTFEs also have a high T_m_.

**Table 1:** Statistical data of DSC and TGA for three PTFE powders.

Sample	T_c_ (℃)	T_m_ (℃)	ΔH_c_ (J/g)	ΔH_m_ (J/g)	X_c_ (%)	T_onset_ (℃)
P5	309.5	329.4	61.6	62.1	75.7	521.2
P220	316.2	330.0	50.1	52.0	63.4	515.0
P500	312.9	326.4	24.8	25.7	31.3	504.4

Figure 3c displays the TGA curves of the three types of PTFE. P500 powder clearly has the lowest initial decomposition temperature of 504.4 ℃ (T_onset_, 5% weight loss); however, P220 and P5 exhibit higher decomposition temperatures than that of P500, 515.0 ℃ and 521.2 ℃, respectively. From the TGA results, it can be concluded that all three PTFEs have high thermal decomposition temperatures and a high temperature resistance. The three types of PTFE have the same molecular structure and chemical elements (C_2_F_4_). Moreover, PTFE is a highly crystalline polymer, and different crystal structures caused by different molecular arrangements result in slightly different thermal properties (T_m_ and stability).

### Morphologies of PBS/PTFE composites

**Figure 4:**
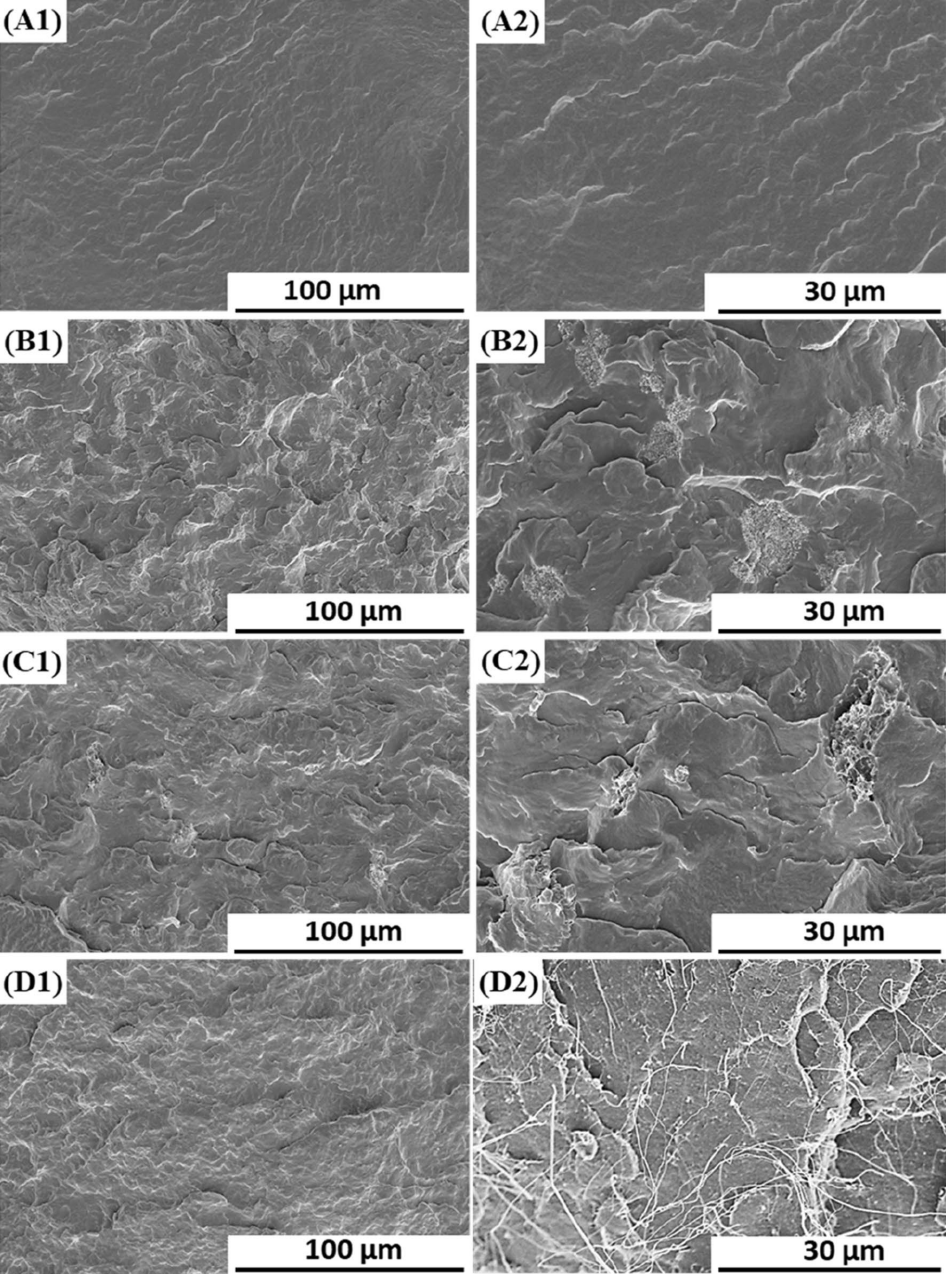
SEM micrographs of the fractured surface of PBS and PBS/PTFE: (**A**) PBS (**B**) PBS/P5 (**C**) PBS/P220 (**D**) PBS/P500.

Figure 4 shows the SEM images of the fracture surface of PBS/PTFE composites. For neat PBS, Fig. 4A shows a homogeneous, smooth and typically brittle fracture surface. However, PBS/PTFE composites (Fig. 4B–D) showed rough surfaces and ductile fractures. Moreover, all types of PTFE had good dispersion in the PBS matrix. P5 and P220 showed the same size as the virgin PTFE powders. Additionally, P5 had superior interface adhesion with the PBS matrix and dispersed well without common agglomeration, demonstrating that P5 had good compatibility with the PBS matrix due to its rough surface character in which the PBS matrix infiltrated the gaps between the tiny nano-PTFE (recall Fig. 2A4). However, the cracks between P220 and the PBS matrix were obvious, indicating poor compatibility, resulting from the better lubricity of P220 itself (smooth surface, recall Fig. 2B).

Interestingly, Fig. 4D shows that almost all P500 was fibrillated and deformed from the original round or oval shape into nanofibrillar structures because of the applied shear and elongational forces during melt blending. Many nanofibers with diameters of 50–200 nm can be observed and interpreted as a result of in situ fibrillation^19,20,25^. Why fibrillation occurred only for P500 has two explanations: the different synthesis process of virgin PTFE particles results in P500 with fibrillation property, or the diameter of P500 (~ 400 μm) is much larger than that of P5 (5–10 μm) and P220 (15–30 μm), resulting in fibrillation more easily under shearing during melt processing. This in situ fibrillation nanocomposite is expected to greatly alter the properties of the PBS matrix.

### X-ray diffraction

**Figure 5:**
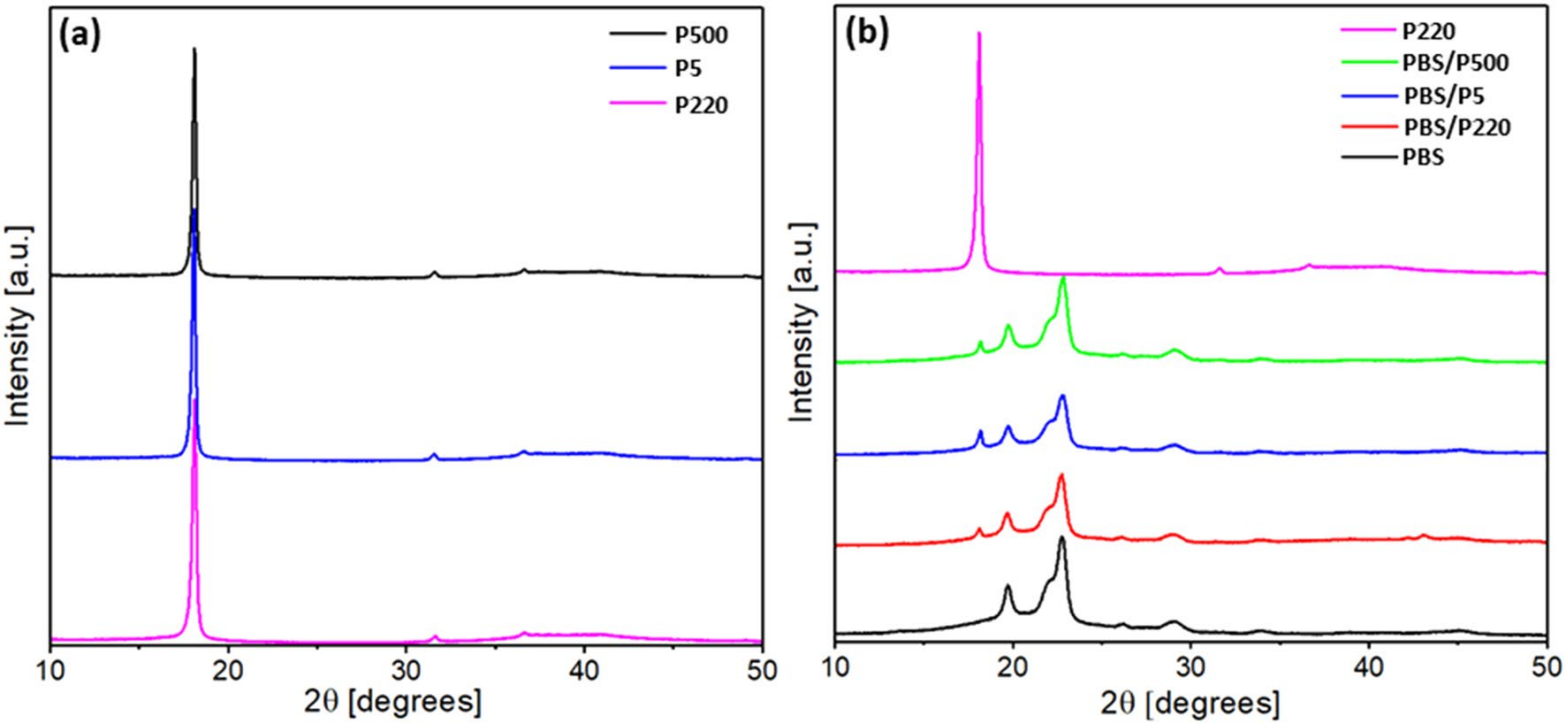
X‐ray diffraction patterns of (**a**) PTFE powders (**b**) PBS and PBS/PTFE composites.

The X-ray diffraction patterns of pure PBS, PTFE and PBS/PTFE composites are shown in Fig. 5. In Fig. 5a, the three PTFE powders showed the same sharp peak at a 2θ of 18.2°. This result indicated that the crystal forms of these three PTFE powders were consistent. In Fig. 5b, PBS exhibited three diffraction peaks at a 2θ of 19.6°, 21.9°, and 22.6°, which correspond to the (020), (021), and (110) crystal planes^10^, respectively. After adding three types of PTFE, the three diffraction peaks at the 2θ of the PBS composites did not shift and appeared as a sharp peak at a 2θ of 18.2° (diffraction spectra of the corresponding PTFE). However, the intensity of the three peaks ((020), (021), and (110)) of the PBS/P500 composites was higher than those of the other two PBS/PTFE composites. The XRD results showed that adding PTFE did not change the crystal form of the PBS matrix but affected the crystallinity.

### Differential scanning calorimetry

**Figure 6:**
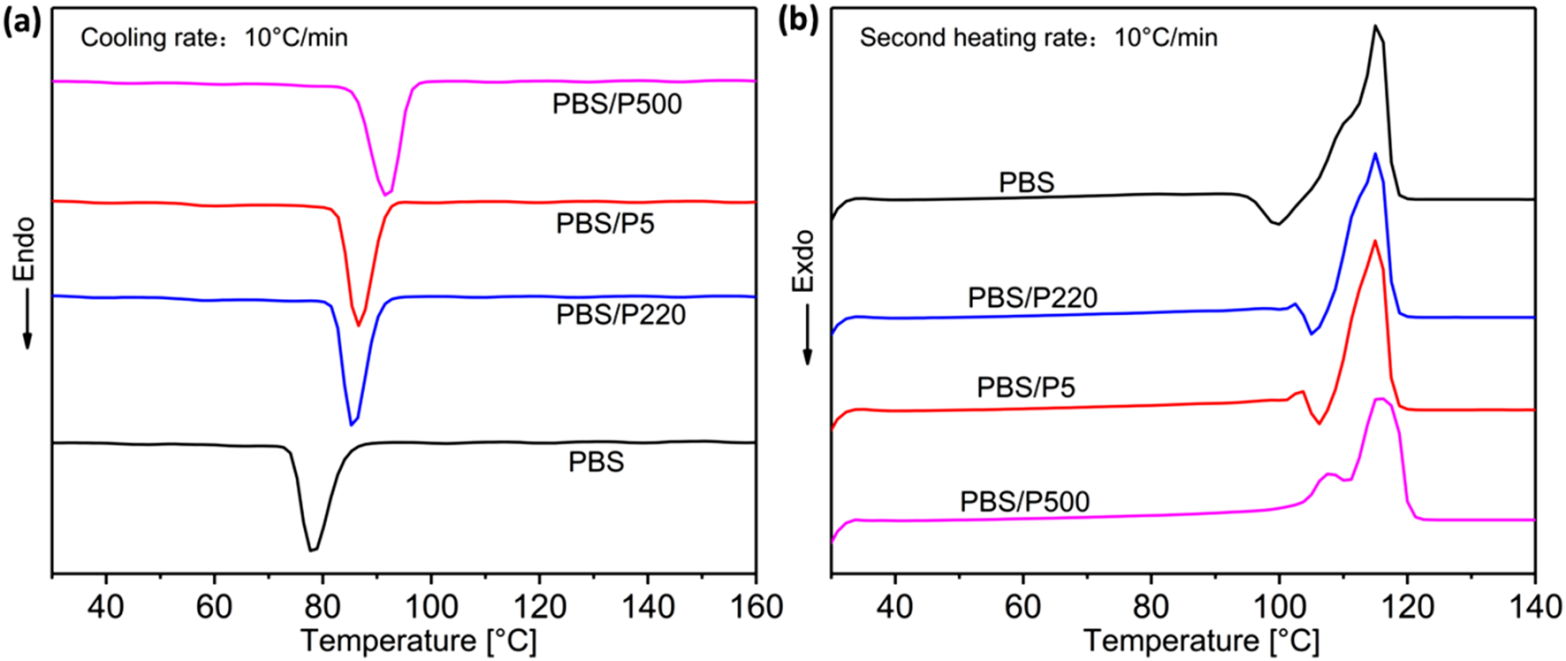
DSC curves of PBS and PBS/PTFE composites: (**a**) cooling and (**b**) second heating.

Figure 6 displays DSC curves of neat PBS and PBS/PTFE composites, and the statistical data are listed in Table 2. The crystallization temperature (T_c_), crystallization enthalpy (ΔH_c_), cold crystallization enthalpy (ΔH_cc_), melting temperature (T_m_) and enthalpy of melting (ΔH_m_) were acquired based on cooling and second heating curves. Figure 6a shows that adding PTFE substantially improved the T_c_ of the PBS matrix as a result of the heterogeneous nucleation of PTFE. P500 had the largest effect (91.8 ℃) because of the in situ nanofiber with a high aspect ratio, which can accelerate the crystallization rate and refine the crystal structure. The T_c_ of the PBS/P5 composite was slightly higher than that of PBS/P220, resulting from the smaller particle size and more uniform distribution of P5, as well as better interface bonding with the matrix. The X_c_ of the three composites exhibited a trend similar to that of T_c_.

**Table 2:** Statistical data of DSC for PBS and PBS/PTFE composites.

Samples	${\mathrm{T}}_{\mathrm{c}}$ (℃)	${\mathrm{T}}_{\mathrm{m}}$ (℃)	$\Delta {\mathrm{H}}_{\mathrm{c}}$ (J/g)	$\Delta {\mathrm{H}}_{\mathrm{m}}$ (J/g)	$\Delta {\mathrm{H}}_{\mathrm{cc}}$ (J/g)	${\mathrm{X}}_{\mathrm{c}}$ (%)
PBS	78.2	113.9	70.2	85.9	10.7	37.6
PBS/P220	85.7	114.5	72.8	81.8	4.5	39.8
PBS/P5	86.1	114.2	76.8	85.1	6.2	40.7
PBS/P500	91.8	116.3	78.2	86.8	–	44.8

As shown in Fig. 6b, for pure PBS, the melting peak appeared at approximately 113.9 ℃. The T_m_ of PBS/P5 and PBS/P220 slightly increased because of the higher X_c_. However, a double melting peak appeared in the DSC curves of the PBS/P500 nanocomposite. This result is interpreted as insufficient crystallization during the cooling process, and a more perfect crystal is reformed by melting recrystallization during the heating process. We know that ΔH_c_ is the crystallization enthalpy and ΔH_m_ is the melting enthalpy. In general, the enthalpy of melting is greater than the enthalpy of crystallization because the melting process in PBS involves the conversion of amorphous to crystalline, i.e., recrystallization in the second heating (Table 2). Figure 6b shows that the absorption peak appears before the melting peak of PBS, PBS/P5 and PBS/P220, which indicates recrystallization. However, PBS/P500 showed a double melting peak, which is interpreted as insufficient crystallization during the cooling process, and a more perfect crystal was reformed by recrystallization during the second heating process.

### Rheological properties

#### Frequency sweep

**Figure 7:**
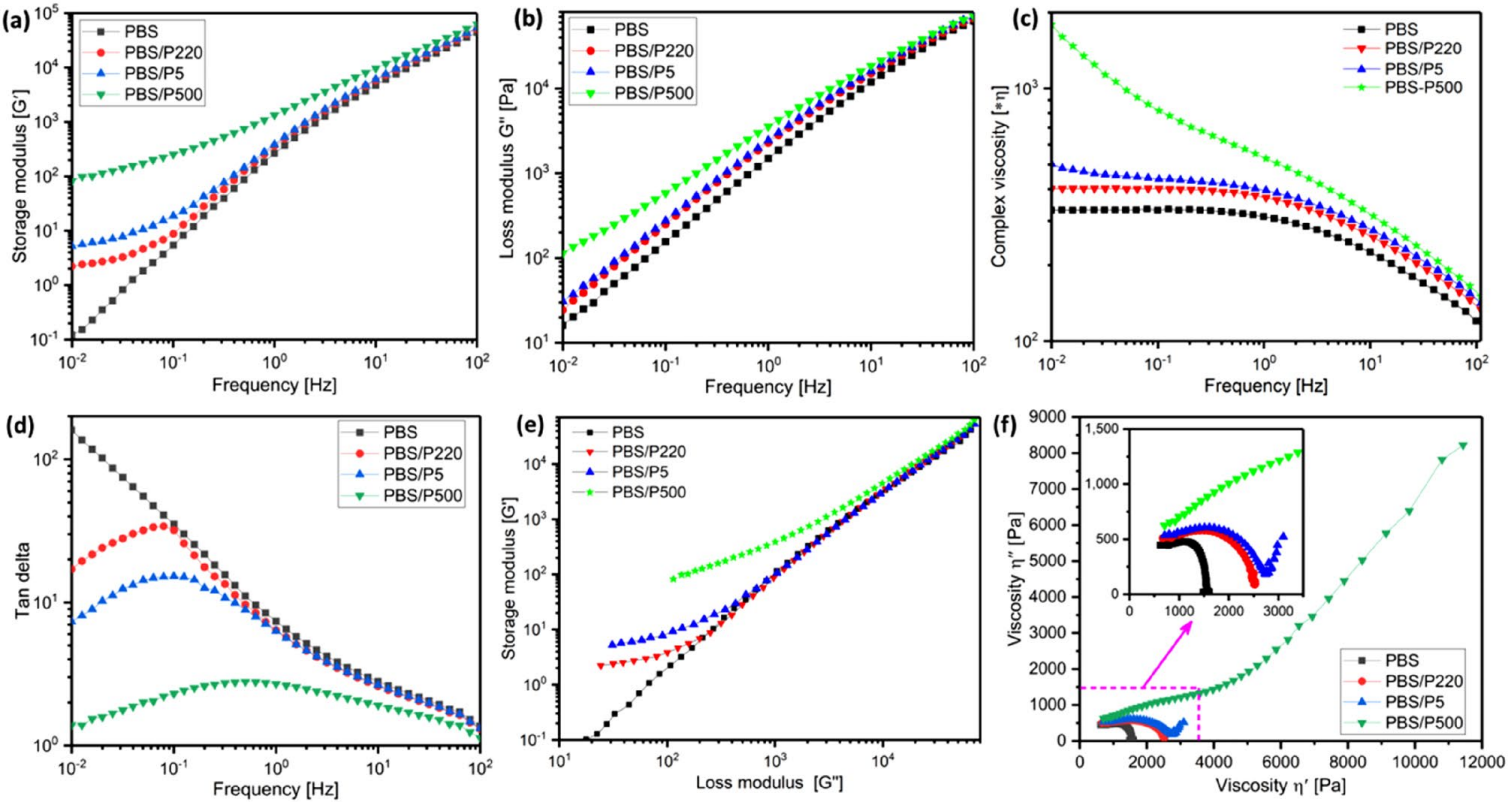
(**a**) Frequency dependence of the storage modulus (G′) and (**b**) loss modulus (G″) at 130 °C, (**c**) frequency dependence of the complex viscosity (η*), (**d**) frequency dependence of the loss tangent (tan δ = G″/G′), (**e**) G′–G″ Han curves, and (**f**) cole–cole curves for neat PBS and PBS/PTFE composites.

The viscoelastic properties of materials are crucial for scCO_2_ foaming performance. In Fig. 7a, neat PBS exhibited a homopolymer rheological behavior^26^. With the addition of PTFE, the dependence of the angular frequency on the storage modulus (G′) tended to weaken. For PBS/PTFE composites, a plateau was clearly observed in the low frequency region, signifying that the rheological behavior had changed from liquid‐like to solid‐like^20^. Thus, the addition of PTFE effectively limited the movement of molecular chains, especially for the in situ fibrillation of P500, which effectively limited the large-scale molecular chain relaxation behavior of the PBS matrix and stored more deformation energy due to the interconnection network structure of the PTFE nanofiber. Figure 7b shows the loss modulus (G″) versus frequency curves of neat PBS and PBS/PTFE composites, which show the same trend as that of G′.

Figure 7c shows the complex viscosity (η*) versus frequency curves of pure PBS and PBS composites at 130 ℃. A typical Newtonian fluid platform appeared in the low-frequency region, and a typical shear thinning behavior appeared in the high-frequency region for pure PBS. After adding P5 and P220, a slightly higher η* was observed. However, the in situ fibrillation nanocomposite of PBS/P500 exhibited strong nonlinear behavior throughout the scanning frequency range, indicating strong interactions between the in situ nanofiber and PBS matrix. In the high-frequency section, the difference in η* between neat PBS and the composites became small because of the extremely low friction coefficient of PTFE itself and the destruction of the nanofiber entanglement network structure of P500.

Figure 7d shows the loss tangent (tan δ) curves of pure PBS and PBS composites. Neat PBS exhibited typical viscoelastic fluid behavior, in which tan δ decreased with increasing frequency throughout the scanning frequency range. However, all three PBS/PTFE composites showed that tan δ increased with the frequency in the low-frequency section, which is a property of physical gel, and tan δ decreased with increasing frequency in the high-frequency section, which is typical of the liquid-like behavior similar to that of pure PBS. Moreover, the transition point (at higher frequencies) was observed in the following order: PBS/P500 > PBS/P5 > PBS/P220, resulting from the higher shear stress, making the transition from solid-like to viscoelastic liquid-like behavior.

The G′–G″ Han^27^ curves of pure PBS and PBS/PTFE composites in Fig. 7e showed a more direct interaction between the PBS matrix and PTFE. The slope of the curve for neat PBS at low frequencies was approximately 2, displaying a homogeneous isotropic system with linear and liquid-like viscoelasticity^28^. In comparison, the slopes of the curves for the PBS/PTFE composites decreased, especially for PBS/P500. The reduced slope indicated a more heterogeneous system. The relaxation behavior of the PBS molecular chain changed because of the addition of PTFE. As the shear frequency increased, the curve of PBS/P220 tended to overlap first with the PBS curve because of the smooth surface structure of P220 itself and the self-lubricating effect. P5 finally tended to overlap, primarily because the entanglement structure gradually disintegrated.

Figure 7f shows the cole–cole curves^29,30^ with local enlarged images of pure PBS and PBS/PTFE composites. Neat PBS showed a single arc, indicating the relaxation time distribution, and the PBS/P220 composite showed a similar curve. However, the curves for the PBS/P5 and PBS/P500 composites had two parts: the first part at low viscosities represented the local relaxation of the PBS chain, and the second part at high viscosities displayed long-term relaxation caused by the restricted PBS chain. In particular, for the PBS/P500 composite, the first part of the arc almost disappeared, and long-term relaxation was dominant. This result clearly demonstrated that the interconnected or entanglement structure of PTFE fibers had been formed, leading to high restriction of PBS chains.

#### Time sweep

**Figure 8:**
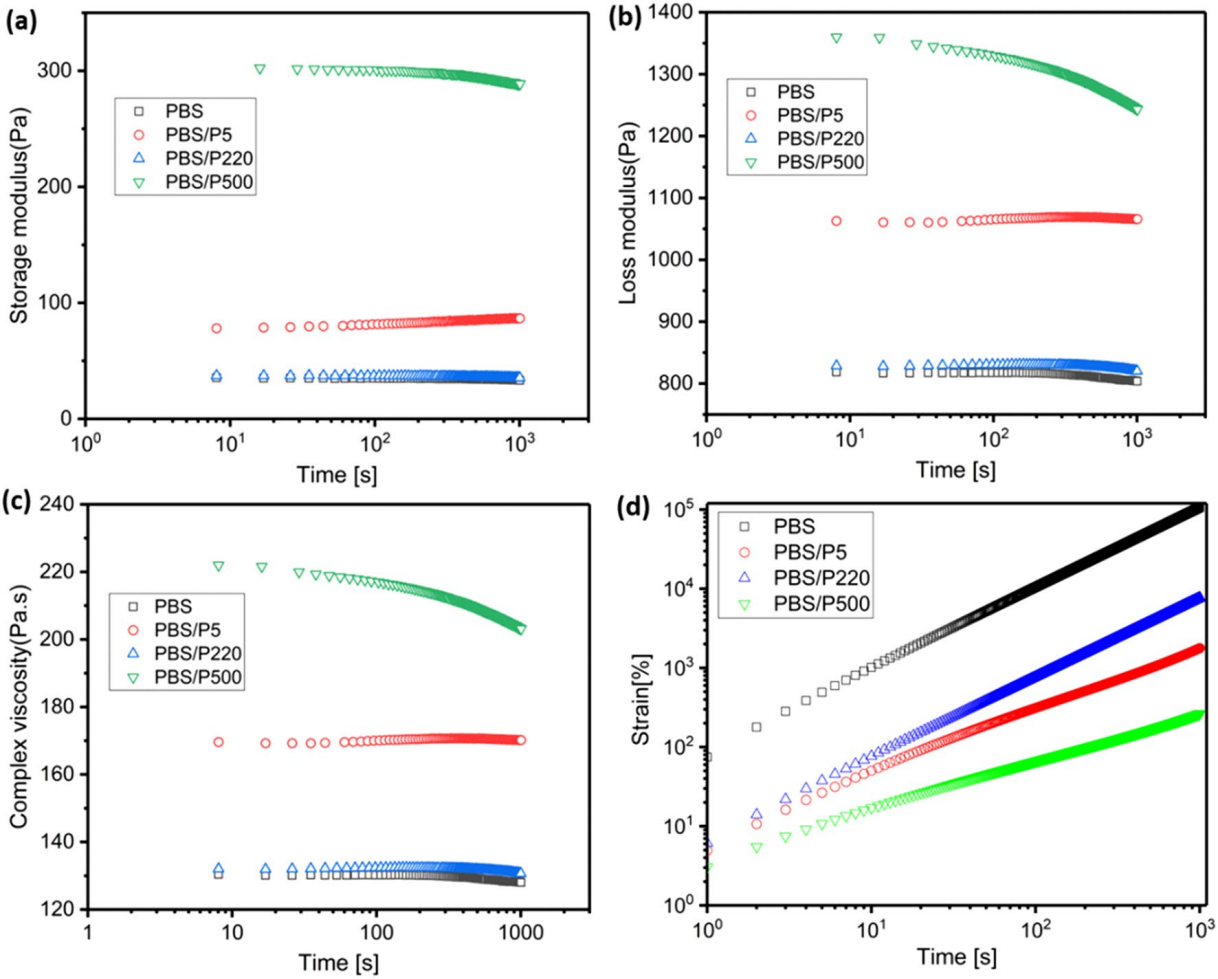
Time sweep curves of rheology: (**a**) storage modulus (G′), (**b**) loss modulus (G″) and (**c**) the complex viscosity (η*); (**d**) creep curves in the molten state of PBS and PBS/PTFE composites.

Figure 8a–c shows the time sweep curves in the linear viscoelastic region. In Fig. 8a, with the addition of P220, no obvious variation tendency was observed compared with that of neat PBS because of the smooth surface of P220 itself (recall Fig. 2B) and the self-lubricating effect. However, PBS/P5 showed a higher G' than that of PBS/P220, resulting from the restrained movement of the PBS molecular chain caused by a smaller particle size and rough surface, as well as good dispersion in the PBS matrix. Exhilaratingly, the G′ of the PBS/P500 nanocomposite further increased because of the existence of an interconnecting PTFE nanofiber network. Figure 8b,c shows that the trend of G″ and η* is similar that of G′. Figure 8d (logarithmic plot) shows that the creep deformation of neat PBS increased linearly with time; however, the strain rates (slopes) of all three PBS/PTFE composites were smaller than that of neat PBS, showing lower strain at the same time. This could be caused by the high viscosity of PTFE and restrained movement of the PBS molecular chain. Among the three types of composites, at the same time, the strain values were as follows: PBS/P500 < PBS/P5 < PBS/P220, which was due to the in situ fibrillated P500 fiber.

### Mechanical properties

**Figure 9:**
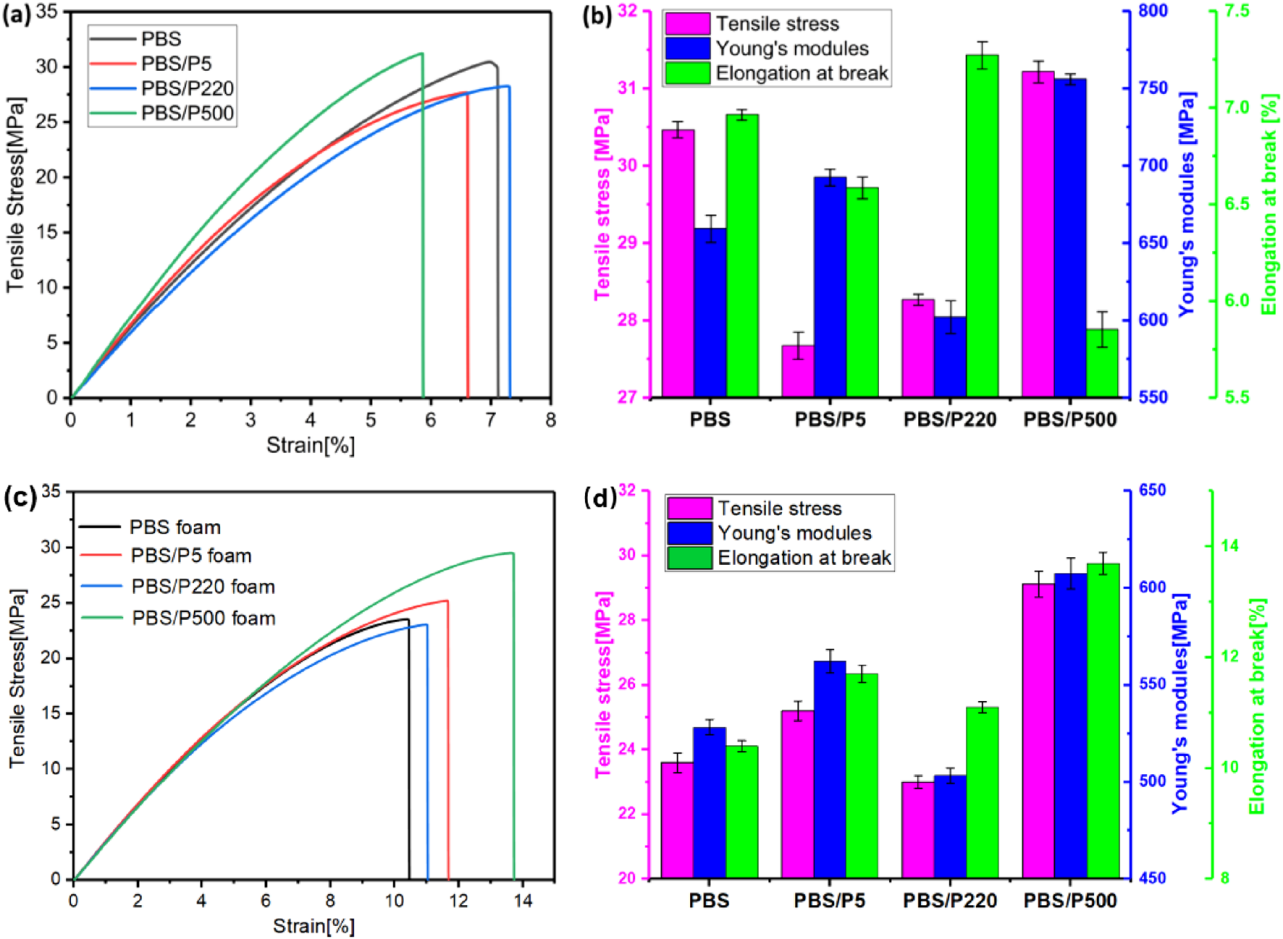
(**a**, **c**) are tensile stress–strain curves; (**b**, **d**) are histograms of the Young's modulus, tensile stress and elongation at the break of solid and foamed PBS and PBS/PTFE composites.

A typical stress–strain curve and histogram of the tensile stress, Young's modulus and elongation at the break for solid and foamed PBS and PBS/PTFE composites are given in Fig. 9a,b. PBS/P220 has the highest elongation at the break because of the smooth surface. However, PBS/P500 has the highest Young’s modulus and tensile stress, which might be due to the in situ fibrillated network structure in the PBS matrix. The Young’s modulus and elongation at the break of PBS/P5 are between those of PBS/P220 and PBS/P500.

**Figure 10:**
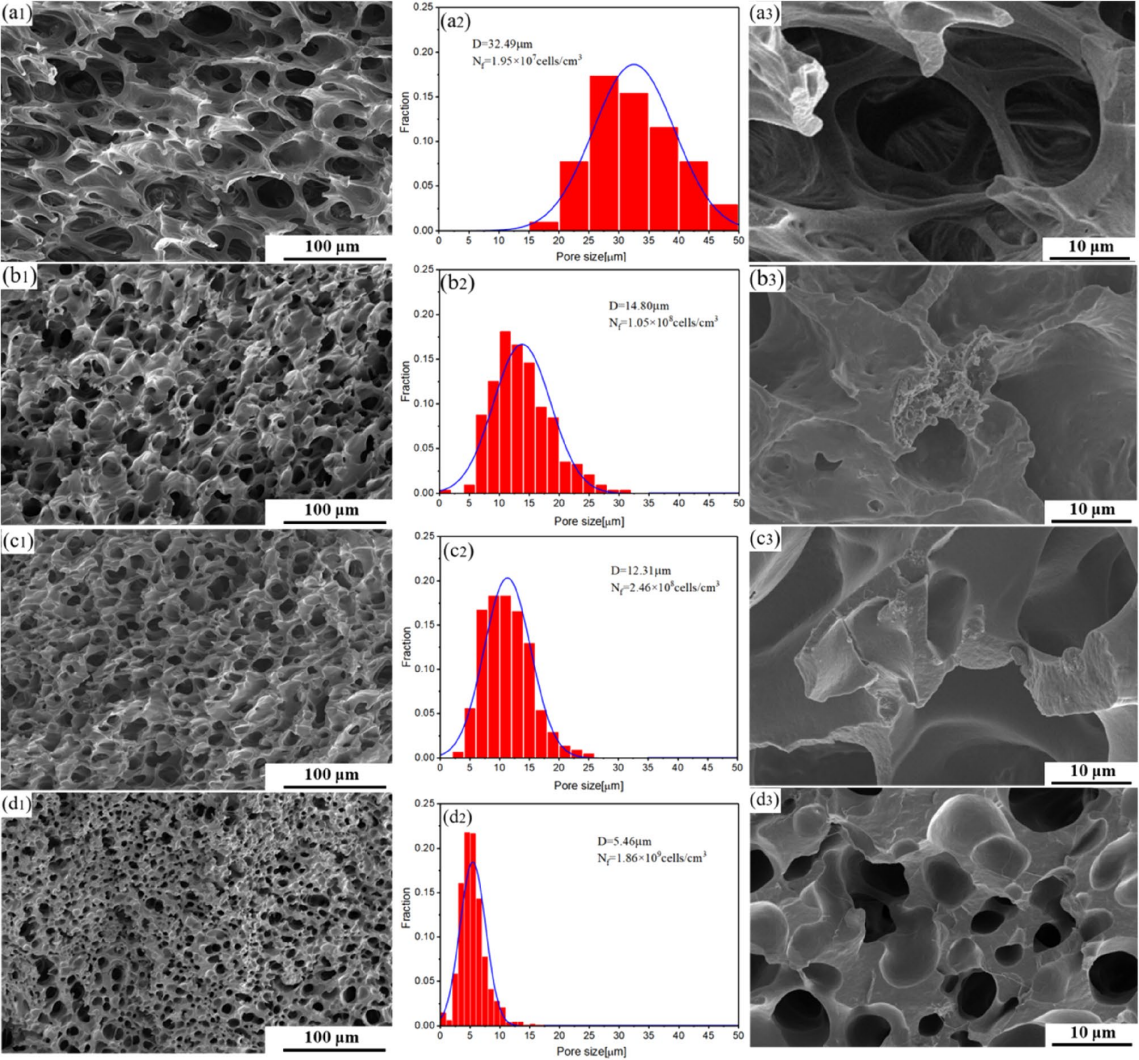
SEM micrographs and size distribution of PBS and PBS/PTFE foams: (**a**) neat PBS, (**b**) PBS/P220, (**c**) PBS/P5, and (**d**) PBS/P500.

The mechanical properties of foams are closely related to the pore structure. Figure 9c,d shows the mechanical properties of PBS/PTFE foams. After foaming, the tensile stress and Young’s modules of neat PBS and PBS/PTFE foams were obviously reduced compared with those of their solid counterparts, resulting from the presence of many imperfect cells after the foaming process (Ref. Fig. 10). In contrast, the elongation at the break of PBS/P500 foams was higher than that of solid PBS/P500, which was due to having the best cell structure and morphology, as well as fibrillated PTFE.

The Young’s modulus of the foams followed the same trend as that of their solid counterparts. The tensile strength obviously increased going from pure PBS foam to PBS/P5 and PBS/P500; however, PBS/P220 foam had less tensile strength than PBS/P5 because of the better cell morphology of PBS/P5 (Ref. Fig. 10). However, the elongation at the break also increased going from pure PBS to PBS/P5 and PBS/P500, and PBS/P500 was the highest in this property among the four types of foams, as a result of having the smallest and most perfect cell structure and fibrillated P500.

### Microcellular morphology

**Figure 11:**
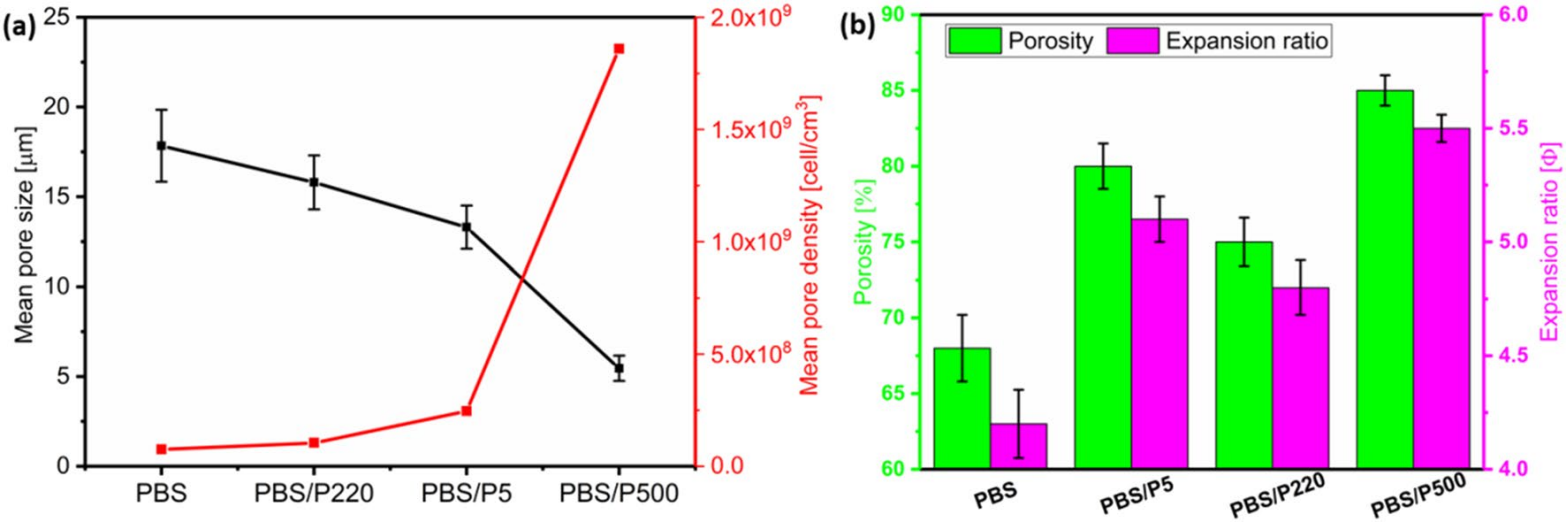
(**a**) Curves of mean pore size and pore density and (**b**) a histogram of the porosity and volume expansion ratio for PBS and the composite foams.

Figure 10 shows the SEM micrographs of neat PBS and PBS/PTFE composite foams, and Fig. 11 summarizes the values of the average cell size, cell density, porosity, and volume expansion ratio of the foams. Figure 10a shows that neat PBS foams presented large and elliptic heterogeneous pore structures, as well as mainly open cell structures. Figure 10c could be shown more clearly (partial enlarged view of Fig. 10a), which was similarly described in other studies^10–15^. This was explained in terms of the poor melt strength of neat PBS. Figure 10b,c shows the PBS/P220 and PBS/P5 foams, which had smaller cell mean diameters, higher cell densities and lower open porosities, signifying a transformation from opening cells to closed cells due to the enhanced melt strength of the PBS matrix^31^. Additionally, in Fig. 10b3,c3, P220 was primarily located at the node of the cell wall, and P5 was uniformly dispersed in the cell wall. PBS/P500 foams (Fig. 10d) were primarily closed cells with a much smaller pore mean diameter (5.46 μm) and much higher pore density (1.86 × 10^9^ cells/cm^3^), which was due to the in situ nanofibrillated fibers greatly improving the melt strength and advancing crystallization^32^. In addition, the high-aspect-ratio nanofibers effectively promoted the heterogeneous nucleation of cells, resulting in the best cell morphology^33^.

**Figure 12:**
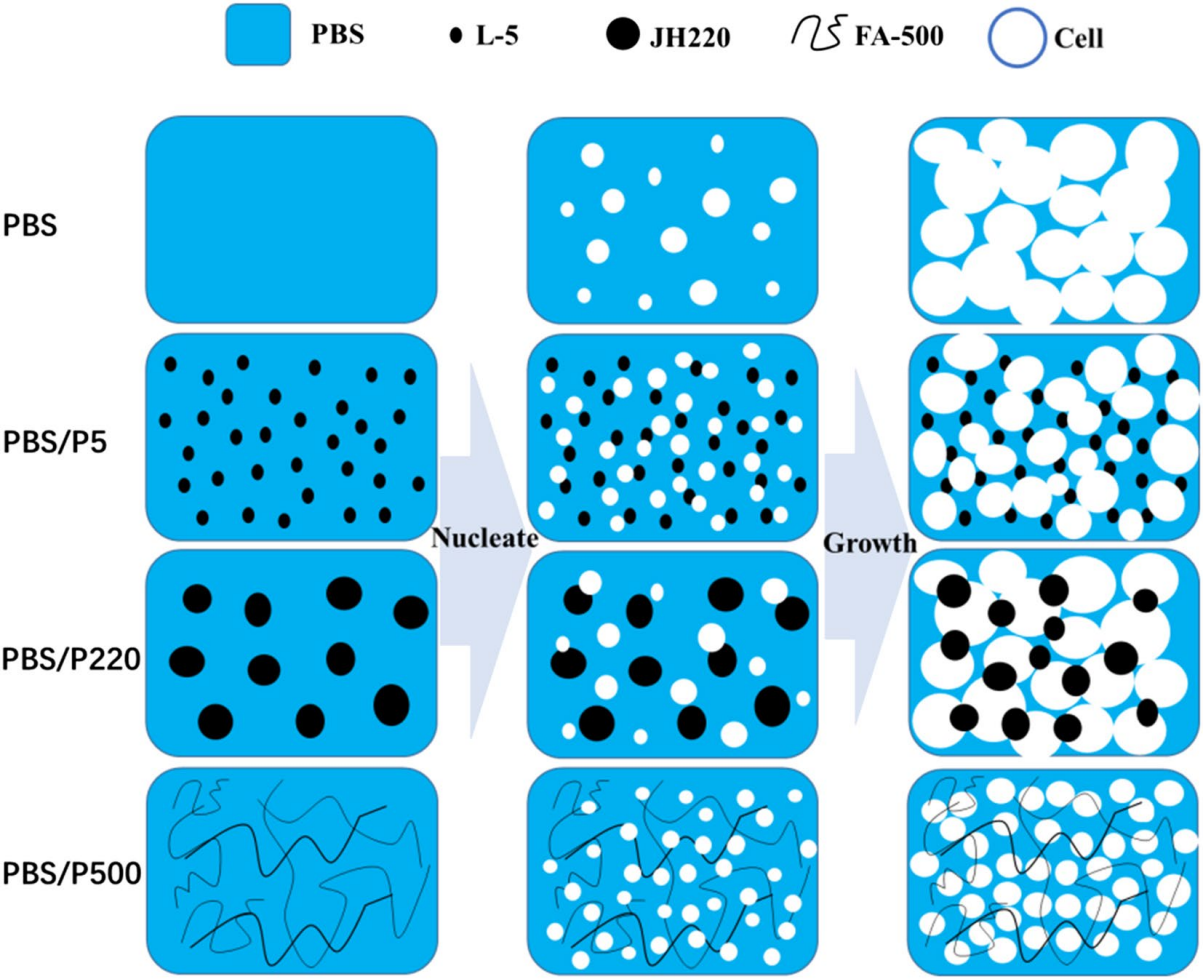
Schematic illustration of fabricating PBS and PBS/PTFE composite foams via scCO_2_ batch foaming.

Figure 12 shows a schematic illustration of fabricating PBS and PBS/PTFE composite foams via scCO_2_ batch foaming. PBS/PTFE composites showed a more significant heterogeneous nucleation effect than that of pure PBS, especially PBS/P500. Consequently, the foamability of PBS was obviously improved by the addition of PTFE powder, and the effect of P500 in situ fibers was the most remarkable.

### Wettability

**Figure 13:**
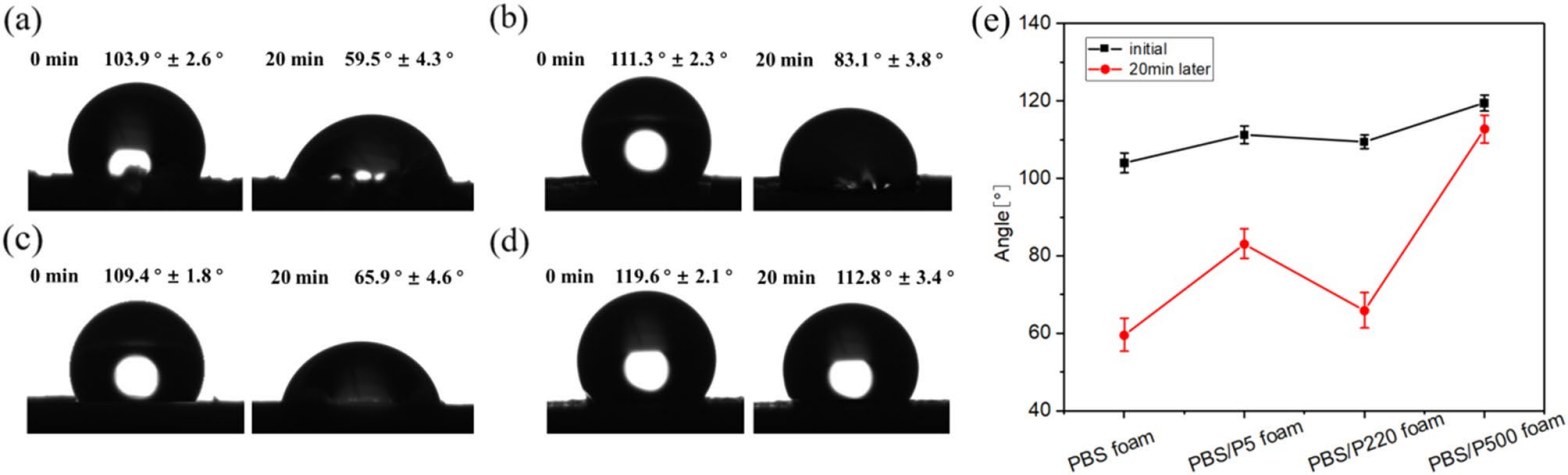
Water contact angle values of PBS and PBS/PTFE composite foams: (**a**) neat PBS, (**b**) PBS/P5, (**c**) PBS/P220, (**d**) PBS/P500, and (**e**) statistical curves.

Figure 13 shows the wettability of the prepared PBS and the composite foams. All foams initially exhibited excellent hydrophobicity, with initial WCA values higher than 90°. However, after 20 min, the WCA values of pure PBS sharply decreased and were almost identical to those of PBS/P5 and PBS/P220, which were lower than 90° and hydrophilic. This was because these foams mainly consisted of large and irregular cells (Ref. Fig. 10), in which water permeated the sample. However, PBS/P500 exhibited hydrophobic properties with a WCA value that was only slightly decreased because of its small and perfect cells. Moreover, PTFE is a highly hydrophobic polymer, and many PTFE nanofibers spread in the matrix (Ref. Figs. 4 and 10).

## Conclusions

The purpose of this work was to study the effect of different types of PTFE polymers on the crystallization properties, rheological properties and foaming behavior of PBS. The results showed that the P5 powder had better compatibility and better dispersion with the PBS matrix than that of P220. P500 underwent fibrillation during melt mixing, forming high aspect ratio nanofibers dispersed in the matrix. PTFE gradually increased the T_c_ of PBS from 78.2 to 91.8 ℃ and the crystallinity from 45.6 to 61.7% without apparent changes in the crystal structure. PBS/PTFE composites exhibited a higher storage modulus, loss modulus, and complex viscosity than those of pure PBS. In particular, the complex viscosity of the PBS/P500 composite increased by an order of magnitude in the low-frequency region, demonstrating that the network structure formed by P500 in situ nanofibers effectively limited the relaxation behavior of the PBS molecular chain. PBS/PTFE composite foams displayed improved foamability with lower cell diameters and higher density. In particular, PBS/P500 foams show closed cells with the smallest pore diameter of 5.46 μm and the highest pore density of 1.86 × 10^9^ cells/cm^3^ by combined action of enhanced melt strength and heterogeneous nucleation promoted by high-aspect-ratio in situ nanofibers. PBS, PBS/P5, and PBS/P220 foams initially were hydrophobic and became hydrophilic after 20 min, but PBS/P500 foam remained hydrophobic. Thus, adding PTFE is an efficient method for fabricating high-performance PBS microcellular foams.
